# The effect of a targeted mobilization program applied after cesarean surgery on care outcomes: a randomized controlled study

**DOI:** 10.1590/1806-9282.20251440

**Published:** 2026-05-11

**Authors:** Birnur Yesildag, Kadriye Aldemir Atmaca, Isa Temur

**Affiliations:** 1 Niğde Ömer Halisdemir University, Zübeyde Hanim Faculty of Health Sciences, Department of Obstetrics and Gynecology Nursing – Niğde, Turkey.; 2 Sivas Cumhuriyet University, Suşehri Health School, Department of Nursing – Sivas, Turkey.; 3 Nigde Omer Halisdemir University, Training and Research Hospital – Niğde, Turkey.

**Keywords:** Gastrointestinal motility, Breastfeeding, Cesarean section, Early ambulation, Pain, Postoperative care

## Abstract

**OBJECTIVE:**

The aim of this study was to determine the effect of a targeted mobilization program applied after cesarean surgery on postoperative recovery outcomes.

**METHODS:**

A randomized controlled trial was conducted with 64 women undergoing elective cesarean section at a training and research hospital in Central Anatolia, Turkey. Participants were randomly assigned to an experimental group (n=32) or a control group (n=32). All women received routine care, while the experimental group additionally received structured mobilization training beginning 6 h postoperatively and continuing for 48 h. Data were collected using the Preliminary Assessment Form, Personal Information Form, Gastrointestinal Function Form, Abdominal Distension Diagnosis Form, Walking Schedule Form, Visual Analog Scale, and Latch, Audible swallowing, Type of nipple, Comfort, and Hold Breastfeeding Diagnosis and Assessment Scale.

**RESULTS:**

Women in the experimental group demonstrated significantly faster gastrointestinal recovery, with shorter times to first gas expulsion and first defecation. Abdominal distension scores at 12, 24, and 48 h postoperatively were consistently lower in the experimental group. Pain scores were also significantly reduced at 24 and 48 h. In addition, the experimental group showed a shorter milk let-down time and higher Latch, Audible swallowing, Type of nipple, Comfort, and Hold breastfeeding scores, indicating better early breastfeeding performance. These differences represent clinically meaningful improvements across multiple recovery indicators.

**CONCLUSION:**

The targeted mobilization program applied after cesarean surgery enhanced overall postoperative recovery by accelerating bowel function, reducing abdominal distension and pain, and improving breastfeeding outcomes. These findings support the integration of structured mobilization strategies into routine post-cesarean care.

## INTRODUCTION

Cesarean section is among the most commonly performed abdominal surgeries worldwide. Although generally considered safe, cesarean delivery is associated with numerous postoperative complications, including pain, bleeding, thromboembolism, and gastrointestinal issues such as nausea, constipation, and abdominal distension^1,2^. These complications can negatively impact maternal recovery, increase hospital stay, and hinder the establishment of early mother–infant interaction.

Early and structured mobilization is a key component of enhanced recovery after surgery (ERAS) protocols. Evidence suggests that timely ambulation promotes wound healing, restores gastrointestinal function, reduces pain and thromboembolic risks, and enhances maternal comfort^3,4^. Although ERAS guidelines are applicable to cesarean delivery, there remains limited evidence on the implementation and effectiveness of structured mobilization programs in this population.

Previous studies have focused on individual interventions such as gum chewing, early feeding, or passive movement; however, there is a lack of standardized, goal-directed mobilization protocols tailored for post-cesarean patients^4,5^. This study aimed to evaluate the effects of a targeted mobilization program initiated in the early postoperative period following cesarean surgery on gastrointestinal recovery, abdominal distension, and pain levels. By providing structured mobilization training and monitoring adherence, this study seeks to contribute evidence-based recommendations for optimizing post-cesarean care.

## METHODS

### Study population and design

This two-arm parallel randomized controlled trial was conducted at a training and research hospital in the Central Anatolia Region of Turkey between August and December 2024. Sample size was determined using pain scores from a similar study^6^, which reported an effect size of 1.00. Based on this, with 95% power and 5% type I error, the minimum required number per group was calculated as 23. To compensate for potential attrition, each group included 32 participants.

Eligibility criteria were age 18–35 years, minimum primary education, singleton low-risk pregnancy, planned spinal anesthesia, absence of psychiatric disorders, and no maternal or neonatal complications. All eligible women provided written informed consent. Block randomization was performed by an independent statistician. Both participants and researchers were blinded to group assignments at the allocation stage. The study was conducted in accordance with the CONSORT guidelines. Ethical approval was obtained from the institutional ethics committee, and the trial was registered with a clinical trial number (Clinical Trials Number: NCT06824337).

### Data collection tools

Data were collected using several structured tools. “The Preliminary Assessment Form,” consisting of nine items, was used to evaluate participants’ eligibility based on inclusion criteria. “The Personal Information Form” collected data on sociodemographic and obstetric characteristics. Postoperative gastrointestinal recovery was assessed using the “Gastrointestinal Function Form,” which recorded the time to first gas and stool passage and was developed based on expert opinion. Abdominal bloating was evaluated at 6, 12, 24, and 48 h postoperatively using the “Abdominal Distension Diagnosis Form,” scored on a 0–10 numeric scale, also developed with expert input. “The Walking Schedule Form” was used to document walking distances on postoperative days 0, 1, and 2. Pain severity was measured using the “Visual Analog Scale (VAS),” a 10 cm horizontal line where 0 indicates “no pain” and 10 indicates “worst imaginable pain”^7^. “The Latch, Audible swallowing, Type of nipple, Comfort, and Hold (LATCH) Breastfeeding Diagnosis and Assessment Scale” is a standardized tool used to evaluate breastfeeding success. The scale consists of five components, each scored from 0 to 2, with a total score ranging from 0 to 10. Higher scores indicate more effective breastfeeding^8^. In the original validation study, the Cronbach’s alpha coefficient was reported as 0.71, indicating acceptable internal consistency. In the present study, the Cronbach’s alpha value was found to be 0.79, demonstrating good reliability within the sample.

### Study implementation

Women admitted for elective cesarean section were first administered the Personal Information Form. Following this, participants in the experimental group received targeted mobilization training. All women in both groups were mobilized 6 h postoperatively in accordance with the hospital protocol. The experimental group followed a structured mobilization program that continued through postoperative days 1 and 2, while the control group received standard care and was asked to self-record daily walking distances.

For the targeted mobilization protocol, women in the experimental group were initially seated on the edge of the bed at 6 h post-surgery, and their vital signs were evaluated. Those with stable vitals were instructed to reset the step-counting app on their smartphones and were assisted to stand. During the first mobilization, women were observed for complications such as hypotension, bleeding, wound issues, or respiratory distress. If no adverse events occurred, they were guided to walk 50 meters. By 24 h postoperatively, participants were expected to complete 1,250 steps. This was increased to 3,000 steps between 24 and 48 h and 4,500 steps between 48 and 72 h, contingent on medical stability. Both groups received routine nursing care, standard pain management, and ambulation at the 6th postoperative hour as required by institutional policy; therefore, the initial timing of mobilization overlapped between the groups. The distinct feature of the targeted mobilization was its structured and goal-directed design, which incorporated clearly defined step targets, progressive increases, and nurse supervision. Unlike the general early ambulation recommended within ERAS protocols, the timing of mobilization could not differ between groups due to institutional restrictions; thus, the differentiation of the intervention rested solely on its structured content rather than earlier mobilization.

At 24 and 48 h after surgery, all participants were assessed using the Postoperative Gastrointestinal Functions Information Form, Postoperative Abdominal Distension Diagnosis Form, the VAS for pain, the LATCH Breastfeeding Diagnosis and Assessment Scale, and the Breastfeeding Knowledge Form. The study was completed with a total of 64 participants, 32 in each group.

### Statistical analysis

SPSS IBM (24.0, IBM Corp., Armonk, NY) program was used to evaluate the data. The normality of the data distribution was examined using kurtosis and skewness values. Descriptive statistical methods included number, percentage, mean, and standard deviation. The chi-square test was used to determine differences between groups, and the independent samples t-test and Mann-Whitney U test were used to compare descriptive characteristics between groups.

### Ethical dimension

Before starting the study, approval was obtained from the Ethics Committee of Nigde Omer Halisdemir University (Decision No: 2024/15-02), and written permission was obtained from the institution where the study was conducted.

## RESULTS

In the study, it was found that 40.6% of the women were between 20 and 25 years old, 51.5% were high school graduates, 51.6% had a moderate economic status, 39% had one pregnancy, 57.8% were undergoing their first cesarean section, and 92.1% had a defecation frequency of at least once a day before surgery. There was no statistically significant difference between the experimental and control groups. No statistically significant difference was found between the experimental and control groups in terms of body mass index (BMI) and preoperative abdominal distension scores.

Women in the experimental group took significantly more steps on postoperative days 1, 2, and 3 compared to the control group, and the effect sizes for these differences were large. The time to first gas expulsion and first defecation was shorter in the experimental group, with statistically significant differences. The effect size for time to first flatus was very large, indicating a clinically meaningful improvement, while the effect size for time to first defecation was moderate (Table 1).

**Table 1:** Comparison of postoperative step counts and gastrointestinal outcome between groups.

Outcome	Experimental group (mean±SD)	Control group (mean±SD)	z/p-value	Cohen’s d	SE	95%CI lower	95%CI upper
Step count (postoperative day 0)	1,371.80±114.95	1,089.06±222.42	-4. 626/**p<0.001**	1.60	0.288	1.03	2.16
Step count (postoperative day 1)	3,154±414.72	2,668.75±323.22	-4.739/**p<0.001**	1.31	0.276	0.76	1.85
Step count (postoperative day 2)	4,531.25±409.11	4,109.37±377.05	-3.886/**p<0.001**	1.07	0.268	0.55	1.60
Time to first flatus (h)	11.65±2.54	15.71±2.08	-5.350/**p<0.001**	-1.75	0.295	-2.33	-1.17
Time to first defecation (h)	71.31±13.68	78.18±12.67	-2.567/**0.010**	-0.52	0.254	-1.02	-0.02

z: Mann-Whitney U test. SD: standard deviation; SE: standard error; CI: confidence interval. The bold values indicate statistically significant differences between the experimental and control groups (p<0.05).

When the postoperative abdominal distension scores of women in the experimental and control groups were compared, no statistically significant difference was found at 6 h postoperatively. At 12, 24, and 48 h, abdominal distension scores in the experimental group were significantly lower, with effect sizes ranging from moderate to very large. Pain scores were similar between the groups at 6 and 12 h, but at 24 and 48 h, pain scores in the experimental group were significantly lower with large effect sizes (Table 2).

**Table 2:** Comparison of abdominal distension severity and pain scores between experimental and control groups.

Postoperative time	Abdominal distension score (mean±SD)		Cohen’s d	SE	95%CI lower	95%CI upper	Pain score (Mean±SD)		Cohen’s d	SE	95%CI lower	95%CI upper
	Experimental group (n=32)	Control group (n=32)					Experimental group (n=32)	Control group (n=32)				
6 h	4.37±0.49	4.50±0.67	-0.22	0.251	-0.71	0.27	5.84±0.91	6.12±0.83	-0.32	0.252	-0.81	0.17
z/p-value	-.533/0.594						-1.324/0.185					
12 h	4.09±0.29	4.34±0.54	-0.58	0.255	-1.08	-0.08	5.46±0.80	5.62±0.75	-0.21	0.251	-0.70	0.29
z/p-value	-2.313/**0.021**						-.899/0.369					
24 h	2.87±0.80	4.62±0.65	-2.40	0.330	-3.05	-1.75	3.87±0.55	4.34±0.48	-0.91	0.263	-1.43	-0.39
z/p-value	-6.485/**p<0.001**						-3.269/**0.001**					
48 h	2.12±0.55	3.21±0.75	-1.66	0.291	-2.23	-1.09	2.75±0.43	3.71±0.85	-1.43	0.281	-1.98	-0.87
z/p-value	-5.295/**p<0.001**						-4.795/**p<0.001**					

z: Mann-Whitney U test. SD: standard deviation; SE: standard error; CI: confidence interval. The bold values indicate statistically significant differences between the experimental and control groups (p<0.05).

The LATCH breastfeeding diagnosis scale scores were significantly higher in the experimental group, with a medium-to-large effect size. It was found that 82.8% of the women initiated breastfeeding between 1 and 3 h after cesarean surgery. The milk let-down process was also shorter in the experimental group, with a large effect size. Additionally, 65.6% of women in the experimental group participated in infant care with assistance (Table 3).

**Table 3:** Comparison of postoperative 24-h breastfeeding characteristics and LATCH scores between groups.

Characteristic	Experimental group (n=32)		Control group (n=32)		Total (n=64)		Statistical analysis	Cohen’s d	SE	95%CI lower	95%CI upper
	n	%	n	%	n	%					
Time to first breastfeeding											
Within 1 h postpartum	2	6.25	3	9.4	5	7.8	X^2^=3.784 p=0.316				
1–3 h postpartum	28	87.5	25	78.1	53	82.8					
4–6 h postpartum	2	6.25	4	12.5	6	9.4					
Milk let-down time (h)	18.96±8.80		29.68±11.89		t=-4.097; **p<0.001**			-1.02	0.266	-1.55	-0.50
Infant care ability											
Independent care	9	28.1	10	31.2	19	29.7	X^2^=1.114 p=0.109				
Assisted care	22	68.8	20	62.5	42	65.6					
Unable to care	1	3.1	2	6.3	3	4.7					
LATCH breastfeeding score	8.40±1.79		6.90±2.00		z=3.154/**0.001**			0.79	0.260	0.28	1.30

χ^2^: Pearson’s chi-square test; t: Independent samples t-test; z: Mann-Whitney U test. SE: standard error; CI: confidence interval. The bold values indicate statistically significant differences between the experimental and control groups (p<0.05).

## DISCUSSION

Gastrointestinal recovery after abdominal surgery is commonly assessed by the time to first gas passage^2^. Early mobilization facilitates this recovery by counteracting anesthesia-related bowel suppression^9^. In our study, women who received targeted mobilization had significantly shorter times to first gas and stool expulsion. These differences demonstrated large effect sizes, indicating clinically meaningful improvements rather than statistically significant differences alone. These results are consistent with previous findings showing that early ambulation, such as walking 5–10 meters, postoperatively enhances bowel function^9,10^. Systematic reviews also confirm the positive effects of mobilization on postoperative gastrointestinal function^11,12^.

Although abdominal distension scores were similar between groups at 6 h, they were significantly lower in the experimental group at subsequent time points. Particularly at 24 and 48 h, very large effect sizes indicate strong clinical impact, suggesting that targeted mobilization meaningfully reduces postoperative abdominal discomfort. This aligns with findings by Dube and Kshirsagar^13^ and Hassan et al.^10^, who reported reduced distension with early mobilization. Similarly, postoperative pain scores at 24 and 48 h were significantly lower in the experimental group, supporting evidence from Alphones and Miranda^4^ and Barai and Vahitha^5^ on the analgesic benefits of early activity. The effect sizes for pain at 24 and 48 h indicate that these reductions are clinically relevant. In this context, a few hours of earlier gastrointestinal recovery and notable reductions in abdominal distension and pain may facilitate earlier mobility, enhanced comfort, and improved discharge readiness.

Breastfeeding difficulties are common after cesarean delivery. The literature indicates that breastfeeding duration and initiation are negatively affected after cesarean section^14,15^. Contributing factors include delayed milk let-down, postoperative pain, limited knowledge, and difficulties with maternal positioning^15^. Acute pain and anesthesia further hinder early initiation within the first hour^16,17,18^. Consistent with previous findings, this study showed low first-hour breastfeeding rates in both groups and prolonged milk let-down in the control group. The reduction in milk let-down time in the experimental group showed a large effect size, suggesting that targeted mobilization has a meaningful impact on lactation physiology. The mean LATCH score at 24 h was higher in the experimental group, indicating greater breastfeeding success. The improvement in LATCH scores represents a moderate-to-large effect and is clinically relevant. While no studies have directly evaluated the effect of mobilization on breastfeeding success, previous research using the same scale reported lower mean scores (6.95–7.21) compared to our findings^14,19^, supporting the role of mobilization in enhancing early breastfeeding outcomes.

### Limitations of the study

There are two important limitations to this study. First, the hospital where the study was conducted did not allow patients to be mobilized before 6 h postoperatively due to institutional policy. Second, the study was conducted in a single center. Additionally, certain potential confounding factors, such as variations in postoperative pain management, differences in patients’ individual motivation levels, and variability in the nursing support they received, could not be fully controlled. These factors may have influenced mobilization performance or breastfeeding outcomes independently of the targeted mobilization program.

## CONCLUSION

The targeted mobilization program applied after cesarean surgery significantly improved postoperative recovery by accelerating the return of bowel function, reducing abdominal distension and pain, and enhancing breastfeeding outcomes. These improvements, supported by large effect sizes, demonstrate that structured and goal-directed mobilization contributes meaningfully to maternal comfort and functional recovery. Integrating such mobilization strategies into routine post-cesarean care may strengthen postoperative management and promote better maternal health outcomes.

## Data Availability

The datasets generated and/or analyzed during the current study are available from the corresponding author upon reasonable request.
